# Primary hepatic origin of a neuroendocrine tumor: A rare case report

**DOI:** 10.1016/j.amsu.2022.104937

**Published:** 2022-11-14

**Authors:** Laila Bouzayan, Ayoub Madani, Samia Malki, Widad Abbou, Imane Skiker, Amal Benani, Rachid Jabi, Mohammed Bouziane

**Affiliations:** a Department of Visceral Surgery and Digestive Oncology A, Mohammed VI University Hospital, Oujda, Morocco; b Faculty of Medicine and Pharmacy, Laboratory of Anatomy, Microsurgery and Surgery Experimental and Medical Simulation (LAMCESM), Mohammed 1st University, Oujda, Morocco; c Department of Anatomopathology, Mohammed VI University Hospital, Oujda, Morocco; d Department of Radiology, Mohammed VI University Hospital, Faculty of Medicine and Pharmacy, Oujda, Morocco

**Keywords:** Liver primary neuroendocrine tumor, Hepatic resection, Case report

## Abstract

**Introduction:**

Neuroendocrine tumors are mainly located in gastrointestinal tract, pancreas and lungs. The primary hepatic origin of neuroendocrine tumors is extremely rare.

**Case presentation:**

A 57-year-old female with a history of cholecystectomy presented to our hospital for right upper abdominal pain lasting for 2 months. Abdominal computed tomography revealed a large exophytic soft-tissue mass in the left liver lobe. Tumor markers were within the normal range. Octreoscan confirmed the primary hepatic origin of neuroendocrine tumor. The patient underwent left hepatic resection. Pathological and immunohistochemical examination of the resected specimen showed a well-differentiated grade 2 neuroendocrine tumor.

**Clinical discussion:**

Primary hepatic neuroendocrine tumors represent rare hepatic tumors. These tumors may occur at any age with an average of 50 years. Diagnosis algorithm includes two key steps: firstly, the confirmation of the endocrine nature of the tumor and secondly the confirmation of its primary nature.

**Conclusion:**

Neuroendocrine tumors are a very rare entity. The primary hepatic location is exceptional. The diagnosis is based on pathological and immunohistochemical examination as well as the result of the octreoscan.

## Highlights

• The primary hepatic origin of neuroendocrine tumors is extremely rare.

## 1 Introduction

Neuroendocrine tumors (NETs) are commonly located in gastrointestinal tract, pancreas and lungs[1], the primary hepatic location is also reported [2]. Neuroendocrine tumors of the liver represent about 0.3% of all NETs [2]. The diagnostic algorithm and therapeutic management are not codified due to their rarity. The treatement is based on surgical resection. This case has been reported following the SCARE criteria [3].

## 2 Observation

**Fig. 1:**
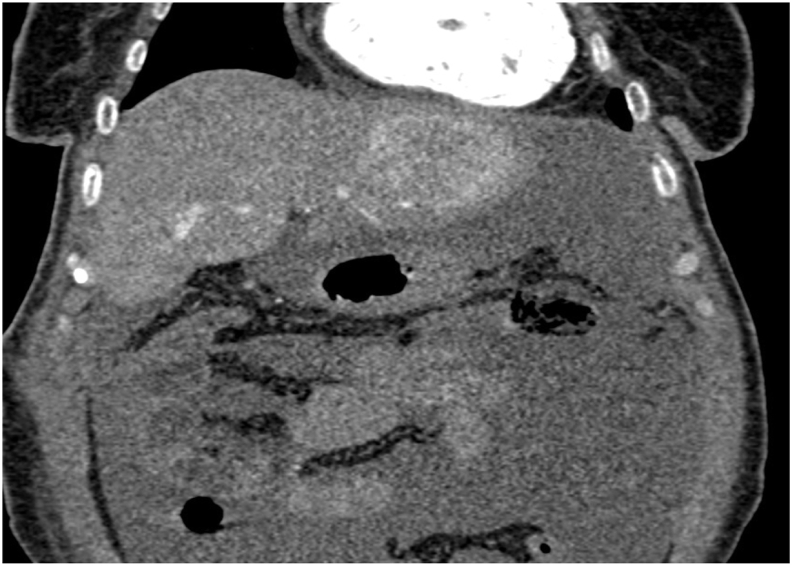
A CT scan revealed a voluminous soft mass of the left liver with exophytic development.

**Fig. 2:**
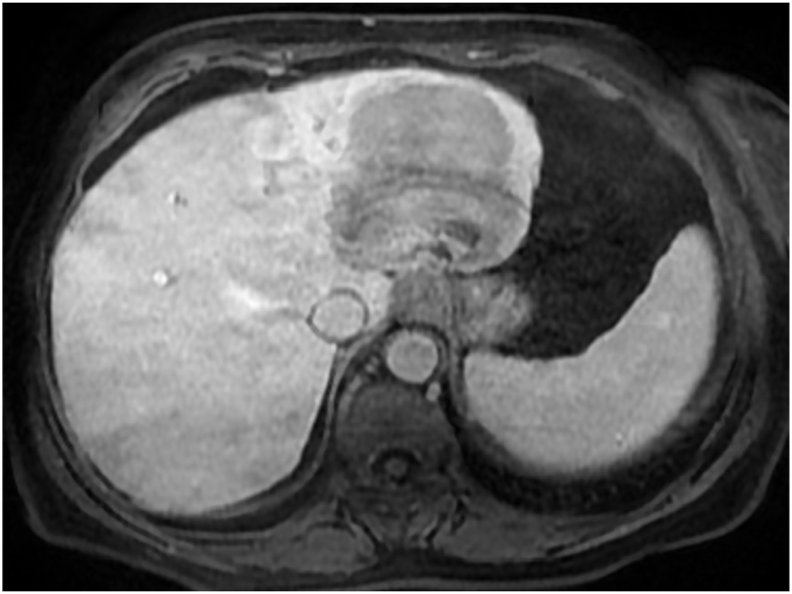
A magnetic resonance imaging showing a voluminous tissue mass of the left liver.

**Fig. 3:**
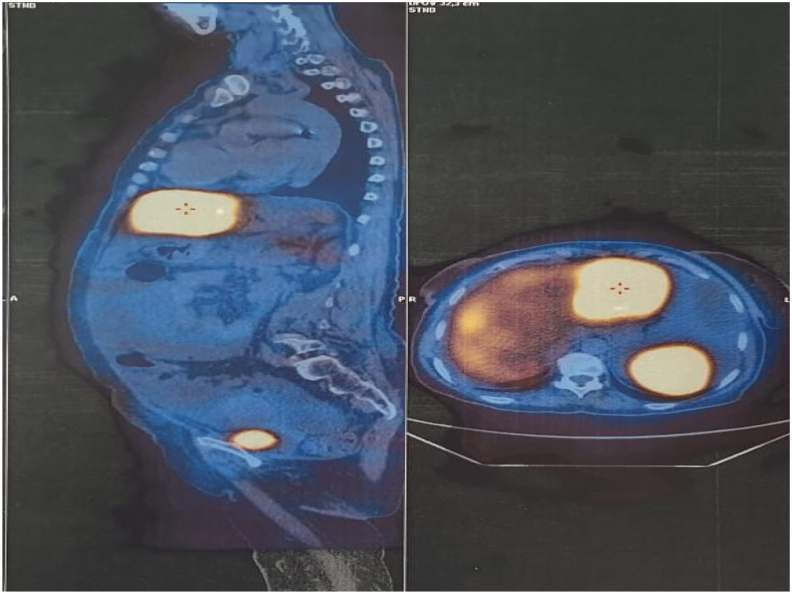
Octreoscan was showed an intense somatostatin receptor hyperfixation area of subtype 2 occupying the hepatic segments II and III evoking the primary origin.

A 57-year-old woman with a history of cholecystectomy ten years ago. She had consulted for upper abdominal pain evolving for two months, without jaundice or other associated signs. The clinical examination was without abnormalities. Abdominal ultrasound examination found a liver mass.A computed tomography(CT)scan revealed a voluminous soft mass of the left liver with exophytic development evoking a malignant lesion in first a hepatocellular carcinoma on healthy liver or a malignant degeneration of an old hepatic adenoma (Fig. 1). A magnetic resonance imaging objectified a voluminous tissue mass of the left liver in T2 hypersignal, T1 isosignal, diffusion hypersignal, enhancing at arterial time and presenting a washout at late time.(Fig. 2). The tumor markers were all normal and the rest of the biological check-up was without abnormalities. A percutaneous liver biopsy was performed, the pathological and immunohistochemical examination was in favor of a well differentiated neuroendocrine tumor of grade 2. In order to search for the primary tumor, a somatostatin receptor scan coupled with a CT scan (octreoscan) was performed and showed an intense somatostatin receptor hyperfixation area of subtype 2 occupying the hepatic segments II and III evoking the primary origin (Fig. 3). The patient underwent surgery. A left liver lobectomy was performed and the patient was discharged on the 10th postoperative day. The pathological and immunohistochemical examination of the resected specimen was in favor of a well differentiated neuroendocrine tumor of grade 2 with mitotic index at 3 and Ki67% estimated at 4.5%. Chromogranin A, synaptophysin and CD56 were positive (Fig. 4a; 4b).

**Fig. 4a:**
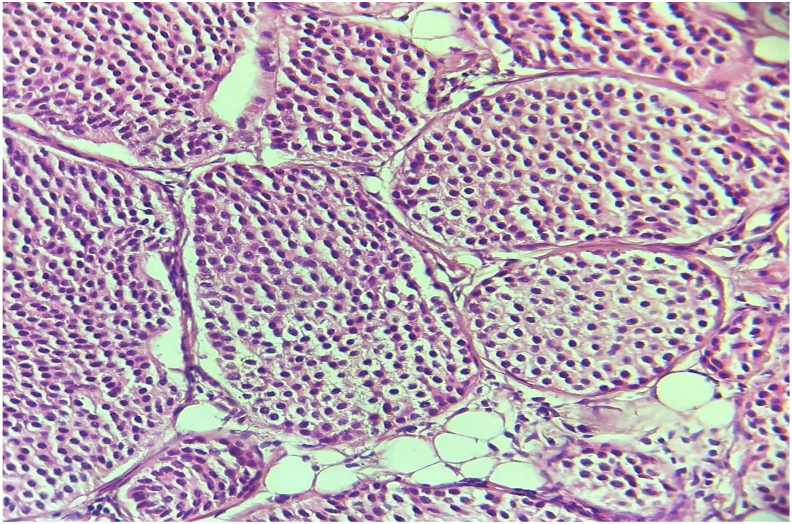
Tumors cells are monomorphic, not atypical (HE, x400).

**Fig. 4b:**
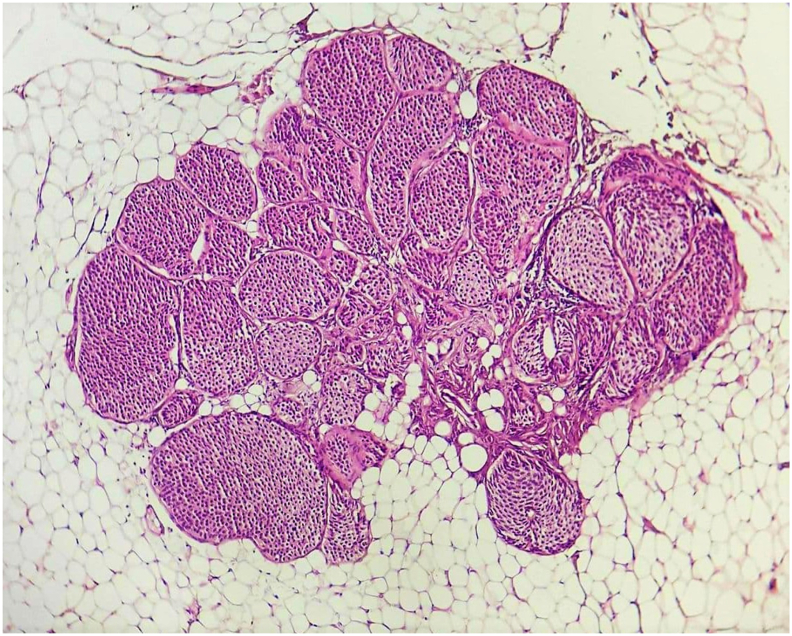
Microphotography showing adipose tissue with a carcinomatous proliferation arranged in nests, surrounded by fine vascularization. (HE, x100).

The patient is currently alive without recurrence at 20 months postoperatively.

## 3 Discussion

NETs originate from neuro-ectodermal cells that migrate from the neural crest from the neural crest to the entire body during embryogenesis [4].

They develop from enterochromaffin cells capable of secreting functional hormones [5]. These tumors are present in the gastrointestinal tract intestinal tract (55%), the pancreas (2%), the bile ducts (1%). However, these cells do not migrate regularly to the liver, which explains the rarity of TNEPF [4,6].

Primary endocrine tumors of the liver are an uncommon pathology. These tumors occur at any age with an average age of 50 years, with a female predominance of 58% [7]. Clinical presentation of neuroendocrine tumors is generally non-specific. In 13% of cases the tumor is completely asymptomatic [7]. Radiologically, the ultrasound imaging is also non-specific; a solid hypo- or hyperechoic mass, associated with a cystic component in 18% of cases [7]. The CT scan often shows a spontaneously hypodense mass associated with a cystic component in 34% of cases, enhanced in the late arterial phase with washout in the portal phase in 26% of cases, thus mimicking a hepatocellular carcinoma HCC [7]. The percutaneous biopsy is performed to demonstrate the neuroendocrine nature of the tumor and the octreoscan is essential in order to eliminate the co-existence of other suspicious lesions and consequently establish the diagnosis of the primary liver origin of the tumor [8,9].

Diagnostic procedure involves two key steps: the first is the confirmation of the neuroendocrine nature of the tumor and the second is the confirmation of its primary nature. The immunohistochemical study remains the only reliable tool to confirm the neuroendocrine nature of the tumor. The specific markers are chromogranin A, NSE and synaptophysin [7,10,11]. The best radiological exam for the detection of neuroendocrine tumors is Octreoscan provided that the tumor expresses somatostatin receptor subtype 2 with a sensitivity of 75–95% [8,11,12]. The gold-standard treatment is, as for any digestive neuroendocrine tumor, surgical resection or hepatectomy, which is the most common treatment, with a 5-year survival rate 74%–78%. The resectability rate is about 86% [7]. Surgical resection depends on the location the tumor, its evolution and its secretory nature.

After surgical resection, the prognosis is generally favorable with a low recurrence rate of 18% [13].

## 4 Conclusion

NETs are a very rare entity and hepatic primary location is also rare. The diagnosis depends on pathological examination and immunohistochemistry as well as the result of the octreoscan. Surgical resection of the tumor remains the gold-standard treatment and long-term prognosis remains favorable.

## Ethical approval

No ethical approval was necessary.

## Sources of funding

The author(s) received no financial support for the research, authorship and/or publication of this article.

## Author contribution

**Dr** Bouzayan Laila: Have written the article, have consulted the patient, and participated in the surgery.

Dr Ayoub Madani: supervised the writing of the manuscript.

Dr Malki Samia: Interpretation of histological data.

Dr Abbou Widad: Interpretation of radiology.

Pr Skiker Imane:(radiology professor): confirm the radiology Interpretation.

Pr Benani Amal (anatomopathology professor): confirm the histological diagnosis.

Pr Jabi Rachid: supervised the writing of manuscript.

Pr Bouziane Mohammed (oncology surgery professor): have supervised the writing of the paper, and has been the leader surgeon of the case.

## Registration of research studies

Not available.

## Guarantor

Dr Bouzayan Laila,Pr BOUZIANE Mohammed.

## Consent of the patient

Written informed consent was obtained from the patient for publication of this case report and accompanying images. A copy of the written consent is available for review by the Editor-in-Chief of this journal on request.

## Provenance and peer review

Not commissioned, externally peer-reviewed.

## Declaration of competing interest

The authors declared no potential conflicts of interest concerning research, authorship, and/or publication of the article.
